# Genomic Approach to Study Floral Development Genes in *Rosa sp.*

**DOI:** 10.1371/journal.pone.0028455

**Published:** 2011-12-14

**Authors:** Annick Dubois, Arnaud Remay, Olivier Raymond, Sandrine Balzergue, Aurélie Chauvet, Marion Maene, Yann Pécrix, Shu-Hua Yang, Julien Jeauffre, Tatiana Thouroude, Véronique Boltz, Marie-Laure Martin-Magniette, Stéphane Janczarski, Fabrice Legeai, Jean-Pierre Renou, Philippe Vergne, Manuel Le Bris, Fabrice Foucher, Mohammed Bendahmane

**Affiliations:** 1 Laboratoire Reproduction et Développement des Plantes, Institut Nationale de la Recherche Agronomique, Centre National de la Recherche Scientifique, Ecole Normale Supérieure, Lyon, France; 2 Unité de Recherche en Génomique Végétale, Institut Nationale de la Recherche Agronomique, Centre National de la Recherche Scientifique, Evry, France; 3 Institut Méditerranéen d'Ecologie et de Paléoécologie, Centre National de la Recherche Scientifique, Université Paul Cézanne-Aix-Marseille III, Marseille, France; 4 UMR Génétique et Horticulture, Institut Nationale de la Recherche Agronomique, Agrocampus Ouest, Université d'Angers, Beaucouzé, France; 5 UMR Bio3P IRISA Equipe Symbiose Campus de Beaulieu, Institut Nationale de la Recherche Agronomique, Rennes, France; Instituto de Biología Molecular y Celular de Plantas, Spain

## Abstract

Cultivated for centuries, the varieties of rose have been selected based on a number of flower traits. Understanding the genetic and molecular basis that contributes to these traits will impact on future improvements for this economically important ornamental plant. In this study, we used scanning electron microscopy and sections of meristems and flowers to establish a precise morphological calendar from early rose flower development stages to senescing flowers. Global gene expression was investigated from floral meristem initiation up to flower senescence in three rose genotypes exhibiting contrasted floral traits including continuous versus once flowering and simple versus double flower architecture, using a newly developed Affymetrix microarray (Rosa1_Affyarray) tool containing sequences representing 4765 unigenes expressed during flower development. Data analyses permitted the identification of genes associated with floral transition, floral organs initiation up to flower senescence. Quantitative real time PCR analyses validated the mRNA accumulation changes observed in microarray hybridizations for a selection of 24 genes expressed at either high or low levels. Our data describe the early flower development stages in *Rosa sp*, the production of a rose microarray and demonstrate its usefulness and reliability to study gene expression during extensive development phases, from the vegetative meristem to the senescent flower.

## Introduction

Roses are widely used as garden ornamental plants and cut flowers. A few flowering traits of roses are essential for the plants commercial value. Examples of these traits are plant architecture, continuous flowering, flower development, function and senescence, scent biosynthesis, reproduction and resistance to biotic and abiotic stresses. However, little is known about the molecular mechanisms that control these traits. This dearth of information limits the scope of rational selection to improve the ornamental plants. During the past decade, using model species such as *Arabidopsis thaliana*, tobacco, *Brachypodium distachyon*, rice or maize, researchers significantly enhanced our understanding of the various aspects of plant development and resistance to biotic and abiotic stresses, and of the molecular and genetic pathways associated with these aspects. However, these model species are not suitable for the studies of other flowering traits such as recurrent blooming, scent production and double flower character. Rose represents an interesting ornamental model species to address some of these aspects.

Cultivated roses have a very ancient history. The two major areas of rose domestication were China and the peri-mediterranean area encompassing part of Europe and Middle East, where *Rosa chinensis* Jacq. and *R. gallica* L. (respectively) were bred and contributed predominantly to the subsequent selection process. Artificial crossing between Asian and European roses gave birth to “modern rose cultivars”. Although testimonies and historical records have documented major crosses that led to modern roses, the genetic basis on which the modern rose cultivars are established is still poorly understood [1]. It has been reported that about 8 to 20 species out of about 200 wild species have contributed to the origin of present cultivars [2], [3], [4].

In *Rosa sp.*, EST sequencing has identified novel genes whose expression is associated with several rose traits [5], [6] such as the scent associated genes O-methyltransferases and alcohol acetyltransferase and floral associated genes [6], [7], [8], [9], [10], [11], [12], [13]. EST sequences were also used to generate a rose DNA microarray comprising 350 selected ESTs [14]. Using this microarray, researchers discovered several novel floral initiation genes and flower scent–related candidate genes (i.e. germacrene D-synthase encoding genes) [15]. However, this array contains only a limited number of sequences that represent genes expressed at late petal development stages.

With publicly available rose gene sequences, we generated a microarray and studied the gene expression throughout floral development, from the initial floral transition to floral senescence. We created an annotated flower EST database corresponding to 4834 genes and used the sequences to develop an Affymetrix microarray. With this microarray, we compared the transcriptome at different floral development stages. We found a good correlation between the microarray data and real time quantitative RT-PCR (qPCR) data for selected genes whose expression coincides with early, mid and late flower development stages. This dataset can help identify new rose genes associated with floral initiation, flower development and senescence.

## Results and Discussion

### Staging the floral transition and flower development in *Rosa sp*

Understanding the genetic basis of flower formation in ornamental plants such as roses is particularly important for future cultivar improvement. We first analyzed the visible morphological modifications during the floral process, from the vegetative meristem to the senescent flower using three rose cultivars, *Rosa wichurana, R. chinensis* cv. Old Blush and *R. x hybrida* cv. Félicité et Perpétue. *Rosa wichurana* and *R. chinensis* cv. Old Blush, two diploid roses, are among the few roses genotypes that were used in the numerous crossings and hybridizations to create the modern roses [2], [16]. For example *R. chinensis* cv. Old Blush contributed major traits, like recurrent flowering and components of the characteristic ‘tea scent’ of modern roses [5], [9], [17], and *R. wichurana* is a non recurrent flowering rose that contributed the climbing trait for some garden roses [17]. The third rose, *R. x hybrida* cv. Félicité et Perpétue (FP) is a cultivated hybrid. These three cultivars were chosen because they have very different flowering habits. For example *R. chinensis* cv. Old Blush was chosen to study floral organogenesis, maturation and senescence, as it flowers all year long in our greenhouse at ENS, Lyon. However, continuing flowering limits our ability to sample enough vegetative meristems for transcriptome analyses. Therefore, to collect sufficient number of meristems, we also chose non recurrent flowering roses, *R. wichurana* and *R. x hybrida* cv. Félicité et Perpétue in greenhouse and field conditions at INRA, Angers.

**Figure 1:**
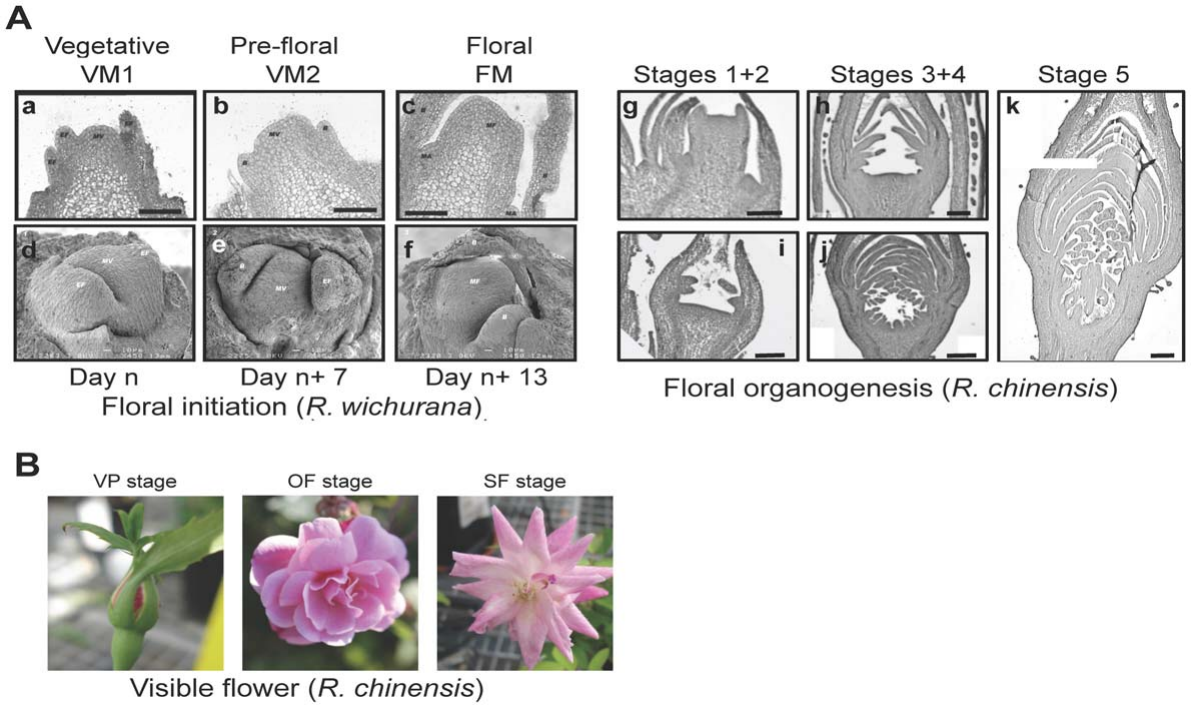
Rose flower development stages. **A**. (a) to (f): Morphology of the floral transition in one-time flowering roses (*R. wichurana*) Schematic representation of the different stages observed during the floral transition in spring is shown in the upper panel from a vegetative meristem (VM) to a floral meristem (FM). a to c: Light microscopy of cross section of meristems. d to f: Environmental scanning electron microscopy images. Black bar: 10 µm. (g) to (k): Rose flower organogenesis stages. Cross sections of floral meristem and young flower buds. Images representing initiation of sepals (stages 1, g), petals (stage 2, i), stamens (stages 3, h) and carpels (stage 4, j). k: hypanthium starts introverting below the floral organs (stages 5). Black bar: 50 µm (g,h,i); 200 µm (j,k). **B**. Visible rose flower stages. Pictures of rose flowers at flower bud with visible petals (stage VP), open flower stage (OF) and senescing Flower stage (SF).

Rose flowers are composed of four organ types arranged in whorls, from the outer to the inner sepals, petals, stamens and carpels. Flower development stages have been determined for model plants such as *A. thaliana* [18]. However, these development stages cannot be directly applied to the rose flower development. In contrast to *A. thaliana* flowers that are composed of four concentric whorls, rose flowers are composed of one whorl of 5 sepals and multiple whorls of petals, of stamens and of carpels. Furthermore, the floral architecture of modern roses differs from that of wild-type roses. For instance, modern rose varieties exhibit double flower character of high number of petals and modified numbers of stamens and carpels, whereas wild-type roses have 5 petals. Scanning electron microscopy (SEM) was used to image floral initiation in *Rosa sp* (Figure 1). Based on these imaging data, we divided the floral initiation process into three stages. After bud outgrowth, the vegetative meristem is dome-shaped and narrow with leaf primordia on its flanks (Stage VM1 for vegetative meristem; Figure 1A, a, d). This structure is typical of a vegetative meristem as previously described [19]. Rapidly, when the new stems have acquired three fully expanded leaves, the meristem enlarges, emerges and leaf primordia are now invisible (Stage VM2, Figure 1A, b, e). We defined this VM2 stage as “pre-floral stage”. Then, the meristem becomes floral characterized by a flat, large and doming structure (Stage FM for floral meristem; Figure 1A, c, f). These morphological changes were similar in the non-recurrent flowering roses, *R. wichurana* and *R. x hybrida* cv. Félicité et Perpétue. Similar enlargement and doming of the meristem were observed during the floral initiation in other related Rosaceae [20].

Sections of floral meristem and young flower buds (Figure 1A, g–k) were used to define the floral organogenesis steps in *R. chinensis* cv. Old Blush. Five morphologically distinct developmental stages were easily distinguished under a dissecting microscope. At flower development stage 1, the floral bud is surrounded by bracts, the floral meristem is flat and five sepal primordia are visible. Floral organs subsequently form following a radial gradient so that the most external organs are the more differentiated. At stage 2, petal primordia are apparent on the flank of the hypanthium. At development stage 3 stamens primordia appear on the flank of the hypanthium while petal primordia continue developing. At stage 4, carpel primordia are the last organs that appear in the center of the hypanthium, while the other organs continue developing. At stage 5, all floral organs are apparent, and the hypanthium starts to sink below the perianth and stamens. During the onward development stages the hypanthium continues to form and the flower becomes clearly visible (Figure 1 B). The four types of floral organs continue developing and flowers start opening (VP stage for visible petals) (Figure 1 B). Then the flower fully opens (OF stage for open flower), and finally senesces (SF stage for senescing flowers).

### Rose EST database creation and Rosa1_Affymetrix custom array design

We collected the available rose genes sequences (ESTs and mRNA) and built a comprehensive database. Using sequence clustering, we generated a dataset comprising 4765 unique sequences (clusters and singletons) and deposited them in http://urgi.versailles.inra.fr/GnpSeq.

For most of the clusters, one representative EST was chosen based the following criteria. Its sequence is larger than 600 nucleotides and preferably corresponding to the 5′ end gene sequence. Because the rose is highly heterozygous, such strategy should prevent using chimerical sequences that might have been obtained during the clustering process. However, 343 clusters did not meet the criteria above. For these 343 clusters, two or more ESTs representing the unique sequence were used. In total, 5175 unique rose EST sequences representing 4765 unique sequences were used for the Rosa1_Affymetrix array design and a total of 6,289 probe sets including Affymetrix control probesets were designed. The arrays were manufactured by Affymetrix (http://www.affymetrix.com).

### Array sequences annotation

We used the Blastx algorithm against the nr database to identify the best protein hits for the 5175 unique rose sequences, and analyzed these results using Blast2go software [21]. 3959 sequences (76.5%) produced a significant match with one or more entry in the database. Among the 3959 sequences, 222 (5.6%) could not be mapped with GO terms and 3737 had at least 1 GO term. For 1439 sequences, full automatic annotations were obtained. Analysis of GO biological process mapping showed that out of these 1439 sequences, 700 (48.6% of mapped sequences) were annotated as involved in primary metabolism processes and only 43 were annotated as putative secondary metabolism genes. 120 sequences (8.33% of mapped sequences) were mapped with the GO:0010468 annotation corresponding to regulation of gene expression. GO molecular function analysis showed that 38 sequences (2.6% of mapped sequences) had putative transcription factor activity (GO:00037000). The complete list of these sequences represented in the array, giving the first Blastx hit, the Blast2go computed annotation and gene ontology, is shown in Table S1. About 23.5% of the rose sequences produced no significant Blast hit in the gene databases. It is likely that the sequences of these genes have diverged far enough to render the annotation difficult. These highly divergent genes may have evolved functions that are be specific to the *Rosa* genus or Rosaceae family and are therefore of particular interest.

### Gene expression associated with rose floral initiation

**Figure 2:**
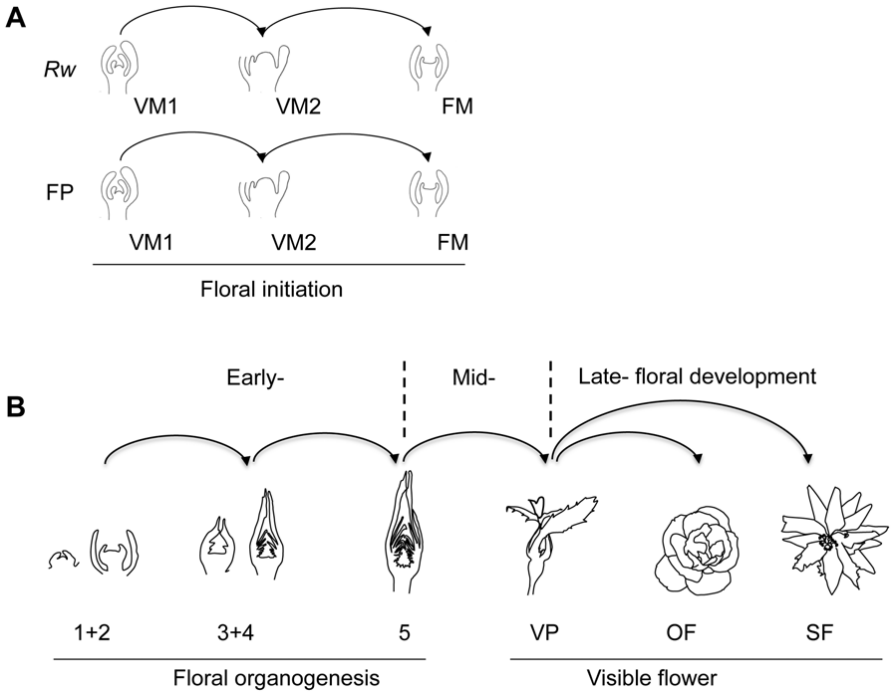
Description of the comparisons performed using micrarrays. **A**. To identify genes associated with floral initiation in *Rosa* using *R. wichurana (Rw)*, *R. x hybrida* cv. Félicité et Perpétue (FP); Comparisons were done in the 2 genotypes; VM1: vegetative meristem stage; VM2: pre-floral meristems; MF: floral meristem. **B**. Schematic representation showing the rose flower development stages from flower organogenesis (stage 1) to onset of senescing flowers (stage SF). Arrows indicates the different transcriptome comparisons. VP: flower bud with visible petals; OF: open flower; SF: Senescing flower.

We analyzed the transcriptomes of *R. wichurana* (*Rw*) and *R. x hybrida* cv. Félicité et Perpétue (FP) during floral initiation. Specifically, we compared vegetative (VM1) to pre-floral (VM2) stages and pre-floral to floral (FM) stages (Figure 2A). Such comparisons can uncover on genes potentially involved in the control of floral initiation. The rationale is that the genes up-regulated between vegetative and pre-floral buds are expected to be putative floral activators. Conversely, genes repressed between vegetative and pre-floral stages are expected to be putative floral inhibitors.

824 genes in *R. wichurana* and 652 genes in *R. x hybrida* cv. Félicité et Perpétue had a dynamic expression pattern between vegetative meristem (VM1) and pre-floral meristem (VM2) (Tables S2 and S3). Between VM1, VM2 and floral meristem (FM) stages, 302 (*Rw*) and 104 (FP) of these genes continued to be differentially expressed. During the VM1 to VM2 transition, 336 (*Rw*) and 301 (FP) genes were up-regulated between vegetative and floral stages, hence they represent candidates associated with floral initiation. 488 (*Rw*) and 351 (FP) genes were down-regulated and they are thus potential floral initiation repressors (Tables S2 and S3). To increase the confidence in the discovery of genes associated with floral induction, the overlapping genes from both datasets (*Rw* and FP) were selected. 258 differentially expressed genes during the VM1 to VM2 transition were common between FP and *Rw* samples. Among these genes, 222 out of 258 (86%) presented the following expression pattern. 131 genes are down-regulated between VM1 and VM2 stages and are thus putative floral repressors (top list in Table 1 and complete list in Table S4A). 91 gene are up-regulated between VM1 and VM2 stages and are thus putative floral activators (top list in Table 1 and complete list in Table S4B). Altogether, these genes are interesting candidates for studying floral initiation in *Rosa* sp.

**Table 1:** Top list of putative floral repressors and activators shared between *R. x. wichurana* and “Félicité et Perpétue”.

		*R. x. wichurana*		“Félicité et Perpétue”	
*Gene*	annotation	*Log(ratio) VM2/WM1*	*Log(ratio) FM/WM2*	*Log(ratio) VM2/WM1*	*Log(ratio) FM/WM2*
***repressors***					
EC58630	0,00	−2,77	−0,95	−1,03	−0,71
BQ104485	(Q5NE18) Formate dehydrogenase	−2,75	1,05	−1,14	−
EC589917	(Q3T923) Fra a 1 allergen (Fra a 1-A allergen)	−2,08	*−0,34*	−4,93	0,20
CF349421	(Q7XHM6) Hypothetical protein OSJNBb0095H08.9	−1,98	1,13	−0,75	*−0,29*
CF349812	(Q8H7G2) Hypothetical protein (Q8H7G2_ARATH)	−1,97	1,22	−1,00	*0,32*
EC587235	0,00	−1,81	0,76	−0,84	*0,46*
EC587845	(Q8L5Z1) Hypothetical protein At1g33810 (Q8L5Z1_ARATH)	−1,79	*0,03*	−1,65	*0,41*
CF349438	(O81644) Villin-2 (VILI2_ARATH)	−1,78	2,21	−0,87	*0,07*
CF349322	(Q1RST0) Peptidase S1 and S6	−1,76	2,40	−2,10	*0,01*
CF349916	(Q564G6) Galactomannan galactosyltransferase	−1,72	0,60	−1,01	*−0,24*
EC587239	0,00	−1,71	1,57	−1,68	*0,36*
BQ105944	ATP-dependent Clp protease ATP-binding subunit	−1,70	1,69	−1,08	*−0,13*
BQ105308	(Q533V0) Phospholipase D alpha (EC 3.1.4.4)	−1,69	1,85	−0,84	*0,31*
CF349664	(Q1S2R3) GIGANTEA protein (Q1S2R3_MEDTR)	−1,68	*−0,13*	−1,27	*0,16*
BI977439	(Q8L553) SCARECROW transcriptional regulator-like	−1,67	1,13	−1,29	*−0,19*
EC586479	0,00	−1,66	1,40	−1,24	*−0,24*
BQ104603	0,00	−1,65	2,31	−1,19	1,00
EC588955	0,00	−1,61	*0,19*	−1,01	*0,67*
BQ106662	(O04136) Homeobox protein knotted-1-like 3 (KNAP3))	−1,58	1,09	−1,02	*−0,17*
CF349422	(Q9SWH0) Plasma membrane proton ATPase	−1,53	1,54	−1,03	*0,13*
BQ106489	0,00	−1,52	0,98	−2,22	−
EC586088	(Q41695) Pectinacetylesterase precursor	−1,48	1,58	−1,32	0,86
EC588764	(Q2HTG1) GTP-binding signal recognition particle SRP54	−1,48	1,09	−1,26	*−0,37*
BI977401	(Q2AAC8) Cysteine proteinase	−1,46	0,60	−1,50	*0,13*
BQ104821	(Q2R3E0) Alpha-mannosidase	−1,46	1,43	−0,74	*−0,11*
BQ103923	(Q84V96) Aldehyde dehydrogenase 1 precursor	−1,46	0,74	−1,64	*0,58*
BQ104041	0,00	−1,45	1,98	−1,47	*0,43*
EC587517	(Q71BZ1) Type-B response regulator (Q71BZ1_CATRO)	−1,42	1,58	−0,70	-
EC588090	0,00	−1,42	-	−2,18	*0,48*
BQ105490	0,00	−1,37	1,83	−5,92	−0,84
BQ103990	(Q8RWI9) Hypothetical protein At3g21090 (Q8RWI9_ARATH)	−1,36	1,32	−1,01	*−0,26*
EC586608	0,00	−1,30	0,91	−0,99	*0,38*
BI978794	(Q05349) Auxin-repressed 12.5 kDa protein (12KD_FRAAN)	−1,30	*0,49*	−2,62	−0,80
CF349291	(Q9SGU9) Similar to O-succinylhomoserine sulfhydrylase	−1,26	0,89	−0,96	*0,01*
EC586448	(Q94BT2) Auxin-induced in root cultures protein 12 precursor	−1,26	*−0,57*	−0,77	−0,72
***activators***					
BI978967	(Q6Z2K3) Putative Avr9/Cf-9 rapidly elicited	1,06	*0,27*	0,75	*−0,04*
BI977621	(Q8L5J6) Expansin 3 (Q8L5J6_MALDO)	1,08	*−0,50*	1,01	0,98
BQ104361	(Q650W6) Putative nucleic acid-binding prot.	1,08	-	1,40	*−0,29*
EC588171	(Q1SZF1) Allergen V5/Tpx-1 related	1,11	−0,75	1,29	0,79
EC586116	0,00	1,12	−1,17	1,14	*0,34*
EC589388	(Q1SHH7) Auxin responsive SAUR protein	1,14	*−0,02*	1,52	*0,47*
BI978946	(Q93Z01) AT5g58730	1,20	*−1,03*	1,55	*−0,17*
RoAGL20	(Q7Y137) MADS-box protein PTM5	1,22	-	0,97	*0,14*
BQ105514	0,00	1,23	*−0,74*	0,72	*−0,02*
BI977348	(Q94AQ7) Hypothetical protein At5g11280	1,23	*0,50*	0,73	*−0,47*
BQ103904	(Q41696) Cysteine protease precursor	1,25	*−2,35*	2,55	*0,20*
EC589855	0,00	1,27	*−0,03*	0,71	*−0,44*
BI978115	(Q84W81) Hypothetical protein At5g49800	1,27	*0,34*	1,85	*−0,57*
EC586690	Q2QXK7) F-box domain, putative	1,31	*−0,91*	1,36	*−0,02*
EC588294	(Q1S0D0) Glyoxalase/bleomycin resistance protein	1,32	*−0,46*	0,77	1,19
EC588783	(Q9LUC1) Putative protein At3g14740	1,34	*0,27*	1,19	*−0,31*
RoAP1a	(Q283Q1) APETALA1 protein	1,38	*−0,40*	2,06	*0,88*
EC587486	0,00	1,42	*−0,62*	1,38	*−0,35*
BI978732	(P32293) Auxin-induced protein 22A	1,47	*−0,36*	1,27	*1,28*
BQ104100	0,00	1,55	*−0,94*	1,49	*1,03*
BQ105108	(O65744) GDP dissociation inhibitor	1,63	*−3,09*	2,13	*0,17*

Log(ratio) of intensities are represented, italicized numbers represent ratios for which the p-value of the Bonferroni test was higher than 0.05. -: no value could be calculated.

**Figure 3:**
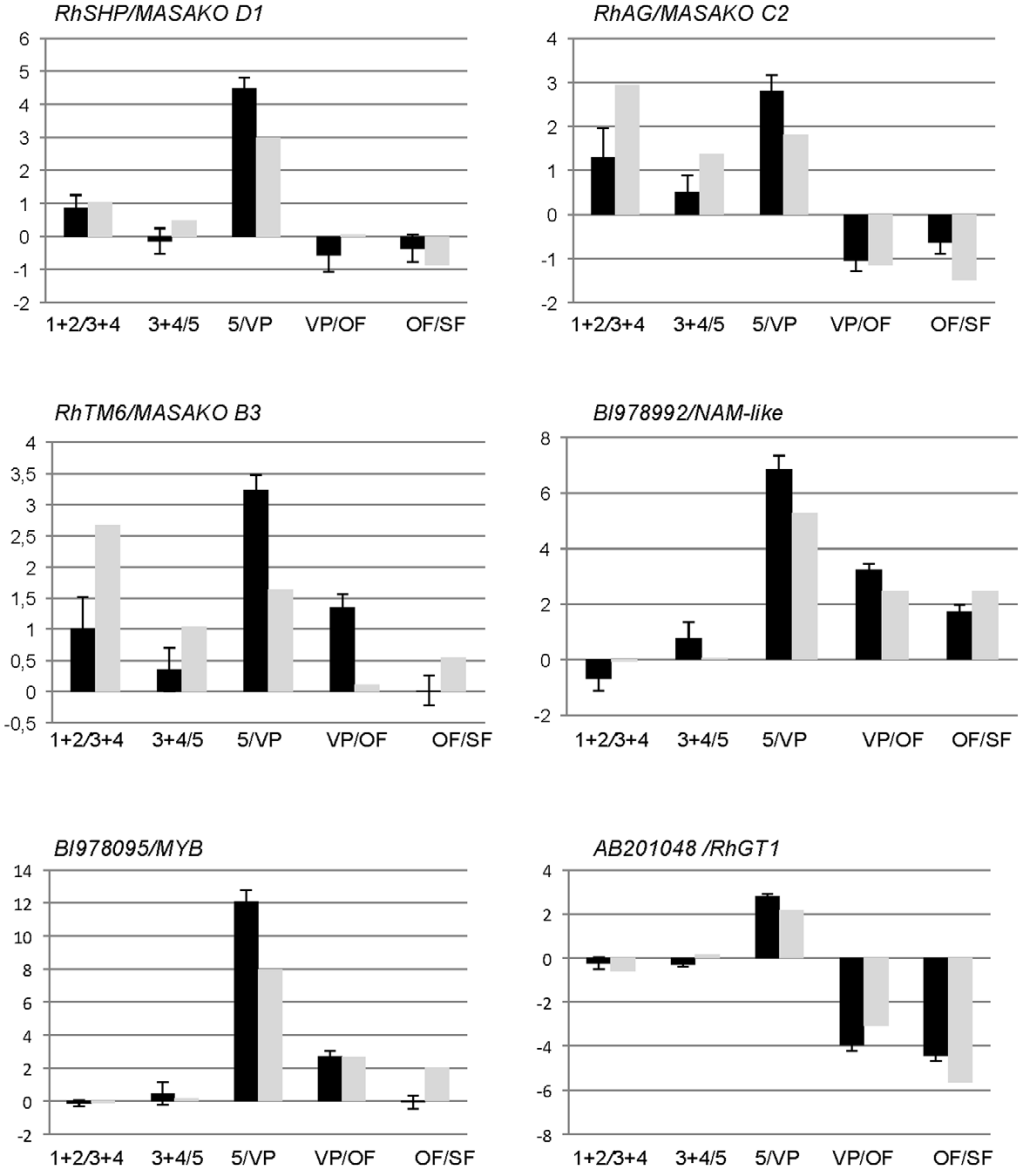
Real time quantitative RT-PCR (qPCR) analysis of six selected differentially expressed genes during rose floral organogenesis, floral opening and senescence in *R. chinensis* cv. Old Blush. qPCR data (black histograms) are compared to the microarray hybridization data (white histograms). Microarray data is presented regardless of Bonferroni test success. Each pair of histograms represent successive comparisons between floral development stages 1+2, 3+4, 5, visible petals (VP), open flower (OF) and senescing flowers (SF).

Among the putative rose floral activators, the expression of the putative rose homologues of *SOC1 (RhSOC1) and APETALA1 (RhAP1)* were induced during the floral initiation both in *R. wichurana* and in *R. x hybrida* cv. Félicité et Perpétue (Tables S2 and S3; Figure 3), in agreement with previously reported data [13]. Therefore, like in *Arabidopsis* [22], [23], in *Rosa sp* the expression of *RhSOC1* and *RhAP1* suggests that these genes may have similar function as floral integrator and floral meristem identity regulator, respectively. Among the genes that were differentially expressed in both roses during floral initiation, six (BI978989, BI978732, BI978794, EC589388, BQ104046, EC586448) showed similarities to genes involved in auxin transport or auxin signalling. Two auxin-repressed homologues (BI978989 and BI978794) were down-regulated and two auxin-induced homologues (BI978732 and BI978794) were up-regulated during the floral initiation process in *Rw* and FP, suggesting dynamic auxin signalling in the rose apex during the floral initiation and the organogenesis of the inflorescence meristems. Auxin and ethylene often interact synergistically [24]–[25]. We found genes involved in ethylene signalling were down-regulated during floral initiation in *Rw* and FP. These genes (EC586386 and AY919867) showed similarities with *EIN* and *EIL* genes [26]. EIN and EIL transcription factors are positive regulators of the ethylene signalling [27]. In *Arabidopsis*, ethylene delayed flowering as *acs* mutant flowered later [28]. In addition, during the floral initiation in *Rw*, two genes showing similarity with ethylene synthesis gene, ACC oxydase (AF441282) and ACC synthase (BQ105189) are down-regulated. Therefore, during the floral initiation, decrease in ethylene production may lead to diminution of EIN/EIL transcription factor and reduction of the ethylene signalling. These expression data suggest that ethylene and auxin may be involved in floral initiation process in rose although further experiments will be necessary to validate these hypotheses.

### Gene expression associated with rose floral development

We harvested six pools of samples corresponding to different flower development stages in *R. chinensis* cv. Old Blush (Figure 1) and compared the transcriptome in successive stages (Figure 2B). We found three distinct groups with common genes (T-test). These groups corresponded to early, mid and late floral development (Figure 2B). A total of 135, 401 and 456 sequences appeared significantly and differentially regulated at least once during early, mid and late flower development stages, respectively.

To validate and evaluate the accuracy of the microarray data, we performed quantitative real-time PCR (qPCR). Twenty four genes were selected from the microarray transcriptomics comparisons based on previous bibliographic reports and/or deregulation levels, then, using qPCR, we further characterized the expression profiles (Figure 3; Figure S1). The correlation between the microarray results and those obtained by qPCR was assessed by calculating the Pearson's product moment correlation coefficient [47], [48] (Table S5). Pearson's correlation coefficient was calculated between each pair of fold change as estimated by microarray and qPCR experiments. The statistical significance of each Pearson's correlation coefficient was assessed using the cor.test routine in R. A global correlation coefficient of 0.858 calculated by the average of every gene was observed. These results indicate that our microarrays are able to detect consistently both low and high fold-changes with high accuracy in different experimental conditions (Table S5).

### Transcriptome analyses during early flower development

135 genes were differentially expressed at during early floral organogenesis. Among these genes, 46 were found differentially expressed between stages 1+2 and 3+4 and 105 genes were differentially expressed between stages 3+4 and 5 (Table 2 and Table S6). An ACC synthase (AY803737) putative homologue was among the highly up-regulated genes between stages 1+2 and 3+4. In *Arabidopsis,* there are nine ACC synthases, many of which are expressed in the flower [29], [30]. The floral organ identity MADS-box encoding genes [31], [32], [33], such as an *APETALA3* homologue (*RhTM6/MASAKOB3 AB055966*, Figure 3), the *AGAMOUS* ortholog (*RhAG, AB025645*, Figure 3), or the rose *PISTILLATA* ortholog (*RhPI*/*MASAKO BP*, *AB038462*), were among the genes whose expression was up-regulated between stages 1+2 and 3+4 or between stages 3+4 and 5. Interestingly, genes that are predicted to have functions in cell wall remodelling, such as putative extracellular lipases (BQ106293, EC586717, EC588243, BI978064, BI977386, BQ105800), xyloglucan endotransglucosylase/hydrolase 2 (*XTH2*, DQ320658) [34], expansins (BI977621, EC589557), putative pectin esterase (BQ105504) and pectate lyase (BQ103887, BQ105987) were up-regulated between stages 3+4 and 5. This result supports the idea that very active cell wall remodeling coincides with the beginning of organ elongation that occurs mainly at stage 5. A putative gibberellin 2-oxidase (BQ105545) was up-regulated early during flower development. In *Arabidopsis*, a similar up-regulation of genes implicated in gibberellins metabolism and signaling have been described at early floral development [35], [36]. In agreement with previously published data, our microarray analysis suggests that gibberellins are important during early floral development of rose plants [13], [37]. Among the genes that showed strong down-regulation between stages 1+2 and 3+4, we found the putative orthologues of *PERIANTHIA* (*PAN*), *AP1* and *SOC1 (AGL20)*. In *Arabidopsis*, *PAN*, *AP1* and *SOC1* are expressed in the floral meristem, but their expression is down-regulated in the subsequent steps during floral organs differentiation [36], [38], [39], [40], hence in agreement with the observed down-regulation of the rose homologues between flower development stages 1+2 and 3+4.

**Table 2:** List of selected floral organogenesis associated genes in *R. chinensis* cv Old Blush.

		*R. chinensis* cv Old Blush	
*Gene*	annotation	Stages 3+4 vs 1+2	Stages 5 vs 3+4
AY803737	Rosa hybrid cultivar 1-aminocyclopropane-1-carboxylase synthase 2 (ACS2)	2,99	−1,21
AB055966	Rosa rugosa MASAKO B3 mRNA for MADS-box protein,	2,67	1,04
AB025645	Rosa rugosa MASAKO C2 mRNA for MADS-box protein,	2,94	1,37
CF349463	(Q1S9M3) Lipase, active site (Q1S9M3_MEDTR)	2,68	-
BI978064	(Q9M8Y5) Putative GDSL-motif lipase/acylhydrolase (Q9M8Y5_ARATH)	*2,10*	1,17
BI977386	(Q9M8Y5) Putative GDSL-motif lipase/acylhydrolase (Q9M8Y5_ARATH)	*1,99*	1,08
EC586717	(Q1S3U7) Lipolytic enzyme, GDS-L (Q1S3U7_MEDTR)	*1,69*	1,16
BQ105800	(Q1SAY6) Lipolytic enzyme, GDSL (Q1SAY6_MEDTR)	2,76	*0,91*
DQ320658	Rosa×borboniana xyloglucan endotransglucosylase/hydrolase 2 (Xth2)	2,55	*0,74*
BI977621	(Q8L5J6) Expansin 3 (Q8L5J6_MALDO)	*−0,89*	1,26
EC589557	(Q9SBT1) Expansin (Q9SBT1_FRAAN)	*0,48*	1,20
BQ105987	(Q94FT6) Pectate lyase B (Fragment) (Q94FT6_FRAAN)	*0,75*	1,27
BQ103887	(Q52PJ2) Ripening-related pectate lyase (Q52PJ2_MANIN)	*1,21*	1,11
BQ105504	(Q7X9B1) Pectinesterase (EC 3.1.1.11) (Q7X9B1_FRAAN)	*1,44*	1,35
BQ105545	(Q4W8C3) Gibberellin 2-oxidase (Q4W8C3_PHAAN)	*0,42*	−1,35
*RoPAN*	(Q9SX27) Putative bZIP transcription factor, PERIANTHIA (Q9SX27_ARATH)	*−1,94*	−2,08
*RoAGL20*	(Q7Y137) POPTM (Q7Y137) MADS-box protein PTM5	−2,77	*−0,03*
*RoAP1b*	(Q2XUP6) MADS-box protein	*−0,98*	−3,15

Log(ratio) of intensities are represented, italicized numbers represent ratios for which the p-value of the Bonferroni test was higher than 0.05.

### Early to late floral development transition

Sequences corresponding to 401 genes were detected as differentially regulated between stages 5 and VP. Among these genes, 233 were down-regulated and 168 were up-regulated (see Table 3 for a selection of genes and Table S7 for full list). Genes that exhibit strong similarities to genes involved in carotene, flavonoid and anthocyanin biosynthesis are up-regulated between stages 5 and VP. Among these genes, putative phytoene synthase (BI979026), zeta carotene desaturase (CF349648), lycopene beta-cyclase (BQ105122) are likely to be involved in carotenoid biosynthesis. The expression of UDP-glucose anthocyanidin-o-glucosyltransferase (AB201048/*RhGT1*), previously involved in anthocyanin synthesis [41], was strongly up-regulated. A similar strong up-regulation was observed for genes encoding putative phenylalanine ammonia-lyase (BQ105227), chalcone synthase (EC587811), flavonol synthase (AB038247) and anthocyanidin synthase (BI977949) (Figure 3). Altogether, these genes are likely good candidates involved in anthocyanins biosynthesis in rose petals.

**Table 3:** List of selected genes associated with early to late flower development in *R. chinensis* cv Old Blush.

		*R. chinensis cv* Old Blush
Gene	annotation	5 *vs* PA
BI978095	(P93474) Myb26	8,00
BI978992	(Q50J79) NAM-like protein	5,28
AB038247	Rosa hybrid cultivar ‘Kardinal’ FLS mRNA for flavonol synthase	4,67
BQ105122	(Q9SEA0) Lycopene beta-cyclase	4,30
EC587811	(Q84UT9) Chalcone synthase	3,27
BQ104100	MYB domain class transcription factor	3,01
AB025643	Rosa rugosa MASAKO D1 mRNA for MADS-box protein.	3,00
CF349648	(Q5W5X6) Zeta-carotene desaturase ZDS2	2,97
BI979026	(Q2VEY1) Putative phytoene synthase	2,89
BI977949	(Q5UL09) Anthocyanidin synthase	2,18
AB201048	RhGT1 UDP-glucose: anthocyanidin 5,3-O-glucosyltransferase,	2,18
CF349636	(Q9ATD1) GHMYB9	2,12
BQ105227	(Q9M567) Phenylalanine ammonia-lyase 2	2,11
EC586028	(Q9SNV1) Cyclin D3a (Fragment)	−2,12
AB201051	RhGT4 mRNA UDP-glucose: flavonol 3-O-glucosyltransferase	−2,18
EC587392	(Q8S342) Putative anthocyanidine rhamnosyl-transferase	−2,45
EC587578	(Q6T2Z6) Cyclin d3	−3,83
EC586734	(Q08733) Aquaporin PIP1.3	−4,52
RhCyc2	(Q9SBQ4) CYCB1-1 protein	−4,65
EC588351	(Q9SBQ4) CYCB1-1 protein	−4,72
EC58848	(P93557) Mitotic cyclin	−4,77
EC589228	(Q94EX2) At1g76540/cyclin dependent kinase	−5,05
EC586517	(Q4JF78) Cyclin-dependent kinase B	−5,26

Log(ratio) of intensities are represented, for all ratios the p-value of the Bonferroni test was lower than 0.05.

Interestingly, genes predicted to encode five putative cyclins (EC586028, EC586517, EC587578, EC588351, and EC588489) and a putative cyclin dependent kinase (EC589228) are strongly down-regulated during floral organ morphogenesis. This down-regulation may reflect the transition from mitotic growth to post-mitotic growth where floral organs grow through cell expansion. Recently, Vanneste *et al*. showed that the transcriptional down-regulation of A2 type cyclins is a direct link between developmental programming and cell-cycle exit in *Arabidopsis thaliana* [42].

Fifteen genes encoding putative transcription factors were up-regulated, while nine were down-regulated. Among the up-regulated transcription factors, we found the putative orthologue of *SHP* (AB025643) [32] and a putative NAC domain protein (BI978992, Figure 3). BI978992 is homologous to *Arabidopsis NAC2*, a gene expressed in ovule integuments. The differential expression of *NAC2* between stages 5 and visible petals (VP) suggests its putatively conserved function with the *Arabidopsis NAC2*. Three putative MYB transcription factors were also up-regulated (CF349636, BQ104100 and BI978095, Figure 3). These rose MYBs may be involved in organ elongation, as they share about 67% protein sequence similarity with AtMYB21, known to be involved in gibberellins/jasmonate-mediated control of stamen filament elongation [43].

### Late floral development

456 genes were differentially regulated at least once during the late phases of floral development, i.e. from visible petal (VP) stage to senescent flower (SF) stage. Most of these genes showed similar expression pattern when we compared stages VP to OF (open flower) or stages VP to SF (See Table 4 for top list, and Table S8 for full data). This result indicates that the transcriptome becomes less dynamic at senescence stages and thus not so many differences are detected when comparing samples OF and SF to the VP sample. Gene ontology analysis showed that among the up-regulated genes, the three GO terms chlorophyll catabolic process, heterocycle catabolic process and cellular nitrogen compound catabolic process were significantly overrepresented as compared to the whole annotated set; the four GO terms nucleus, macromolecule biosynthetic process, intracellular non-membrane-bounded organelle and ribonucleoprotein complex were underrepresented. We could identify two genes encoding stay-green protein homologues (BI978267 and BQ106457) that are strongly up-regulated upon petal elongation and remain highly expressed throughout the final petal senescing process. Stay-green proteins have a major role in chlorophyll and photosynthetic pigments degradation and have been repeatedly described to be associated with the processes of fruit ripening and organ senescence [44]. Surprisingly, no gene related to ethylene biosynthesis or signaling was detected as differentially expressed during late floral development. However the *RbXTH1* and *RbEXPA1* genes, both induced during ethylene-triggered and field abscission [34], [45], were strongly up-regulated between VP and OF stages and remained as such in senescing flowers. Among the down-regulated genes, the two GO terms protein metabolic process and plasma membrane were underrepresented as compared to the whole set (whole microarray GO terms) and the eight GO terms acyltransferase activity, acyl-carrier-protein biosynthetic process, acyl carrier activity, cellular carbohydrate metabolic process, polysaccharide metabolic process, fatty acid biosynthetic process, lipase activity and defense response to fungus were overrepresented (Table 5). The enrichment in the latter set may represent a slowdown of general metabolic pathways at the onset of flower senescence. Similar results were reported in *A. thaliana* during organs senescence where a down-regulation of the photosynthetic machinery accompanied by a reduction in expression of many cell wall biosynthetic genes reflecting a cessation of growth during senescence [46].

**Table 4:** List of selected floral maturation and senescence associated genes in *R. chinensis* cv Old Blush.

		*R. chinensis* cv Old Blush	
Gene	annotation	PA *vs* FE	PA *vs* FS
BI977502	Brassinosteroid-regulated protein BRU1 precursor	8,82	8,79
BI978598	Early light-induced protein	8,38	7,99
EC587309	0,00	7,70	5,82
BI977376	Putative zinc finger protein At1g68190	7,35	7,13
BI978143	0,00	6,61	7,00
EC587486	0,00	6,31	4,81
BI978750	0,00	6,30	5,55
BI978596	Hypothetical protein	6,29	6,32
BQ104828	0,00	6,24	5,51
BQ105724	0,00	6,09	5,65
EC586975	Glycosyltransferase NTGT5a	6,07	6,28
BQ106572	0,00	6,02	5,87
BI977926	18.5 kDa class I heat shock protein	5,83	-
EC588495	0,00	5,60	5,54
BI978508	Hypothetical protein	5,51	-
BQ104475	0,00	5,50	6,25
BQ103870	0,00	5,26	5,12
BI977873	Hypothetical protein At5g63130	5,25	5,18
BQ103973	Tryptophan synthase alpha chain	5,24	4,61
BI977634	Aux/IAA protein	5,21	5,20
BQ105490	0,00	5,07	5,85
BQ106477	Protein WUSCHEL-like	5,03	5,54
BQ106330	0,00	5,02	5,22
BQ106091	Hypothetical protein At2g42570	4,91	4,52
BI977302	Bzip transcription factor	4,82	4,83
BI978926	AT5g11580	4,81	5,46
CF349316	Putative NADH dehydrogenase	4,81	5,01
EC589818	Putative calmodulin-related protein	4,80	-
BI978132	0,00	4,74	4,55
BQ105726	Expansin-like protein	4,73	3,90
BQ104701	0,00	4,68	4,81
EC586683	0,00	4,68	5,52
DQ320657	*Rosa×borboniana* expansin protein (ExpA1) mRNA	4,17	4,38
EC589229	Probable xyloglucan endotransglucosylase/hydrolase protein 8 precursor	3,20	-
BI978267	Senescence-inducible chloroplast stay-green protein 1	2,71	2,42
BQ106457	Senescence-inducible chloroplast stay-green protein 2	-	2,34
BQ104919	Pectate lyase	−4,04	−6,18
EC588897	Laccase	−4,04	−4,86
EC588483	Alpha-D-xylosidase precursor	−4,06	−3,72
EC587152	0,00	−4,08	−4,46
EC589569	Serine carboxypeptidase, putative	−4,10	−3,48
EC586717	Lipolytic enzyme, GDSL	−4,13	−2,59
EC587284	RNA-binding region RNP-1	−4,15	−3,42
BI977461	0,00	−4,21	−4,40
EC586015	Cold-regulated LTCOR12	−4,22	−4,44
BI978116	Putative cell-wall P4 protein	−4,24	-
BI977348	Hypothetical protein At5g11280	−4,27	−4,99
BQ106043	Isoamylase isoform 3	−4,27	−4,01
EC589137	Putative alpha-glucosidase	−4,33	−4,07
EC587785	0,00	−4,37	−3,91
EC586984	Putative beta-expansin	−4,38	−4,99
CF349422	Plasma membrane proton ATPase	−4,41	−3,64
BI977751	0,00	−4,49	−4,43
EC589098	Phosphoethanolamine N-methyltransferase 1	−4,49	−5,79
BQ106293	GDSL-motif lipase/hydrolase-like protein	−4,51	−6,32
CF349724	Glucosyltransferase-like protein	−4,74	−4,10
BQ106328	Pathogenesis-related transcriptional factor and ERF	−5,10	-
EC586884	Proline-rich protein APG-like	−5,11	−3,72
CF349712	Senescence-inducible gene protein	−5,12	−5,47
BI978135	Plant lipid transfer protein/Par allergen	−5,16	−7,04
CF349692	Putative alcohol oxidase	−5,17	−6,66
EC588080	Hypothetical protein (At2g35760/T20F21.5)	−5,32	−5,20
BI977262	Putative lipase	−5,48	−6,04
CF349791	Globulin-like protein (Fragment)	−5,67	−5,53
AB121046	phloroglucinol O-methyltransferase, complete cds	−6,13	−5,06
BI978206	0,00	−8,51	-
BI978064	Putative GDSL-motif lipase/acylhydrolase	−10,47	−10,51
BI977386	Putative GDSL-motif lipase/acylhydrolase	−11,60	−11,42

Log(ratio) of intensities are represented, for all ratios the p-value of the Bonferroni test was lower than 0.05.

**Table 5:** Gossip analysis of GO terms enrichment in late flower development dataset (genes that are differentially expressed at least once during floral maturation and senescence).

	GO Term	Name	FDR	FWER	single test p-Value	# in test group	# in reference group	# non annoted test	# non annoted reference group	Over/Under
Late floral development upregulated genes	GO:0005634	nucleus	0.0	0.0	0.012	0	107	56	1290	under
	GO:0009059	macromolecule biosynthetic process	0.0	0.0	0.028	0	88	56	1309	under
	GO:0043232	intracellular non-membrane-bounded organelle	0.0	0.0	0.030	0	86	56	1311	under
	GO:0030529	ribonucleoprotein complex	0.0	0.0	0.030	0	84	56	1313	under
	GO:0015996	chlorophyll catabolic process	0.008	0.008	5.43E-5	3	0	53	1397	over
	GO:0046700	heterocycle catabolic process	0.028	0.062	5.13E-4	3	2	53	1395	over
	GO:0044270	cellular nitrogen compound catabolic process	0.028	0.062	5.13E-4	3	2	53	1395	over
Late floral development downregulated genes	GO:0019538	protein metabolic process	0.0	0.0	0.009	5	225	73	1150	under
	GO:0005886	plasma membrane	0.012	0.006	0.013	4	192	74	1183	under
	GO:0008415	acyltransferase activity	0.016	0.025	1.62E-4	7	17	71	1358	over
	GO:0042967	acyl-carrier-protein biosynthetic process	0.016	0.028	2.15E-4	7	18	71	1357	over
	GO:0000036	acyl carrier activity	0.027	0.079	5.73E-4	3	1	75	1374	over
	GO:0044262	cellular carbohydrate metabolic process	0.027	0.084	6.73E-4	15	99	63	1276	over
	GO:0005976	polysaccharide metabolic process	0.027	0.086	7.08E-4	10	48	68	1327	over

The reference group that was used corresponds to the full annotated sequences (sequences with GO terms) of the microarray.

### Conclusions

We established a calendar of the floral initiation and development for the rose and developed a rose microarray that harbors sequence from genes expressed during the floral transition and whole floral development process in *Rosa sp*, from initiation up to senescing flowers. This microarray and the floral development calendar were successfully used to identify genes whose expression correlated with different flower development stages.These multiple datasets represent an extensive study of rose floral development. This resource can be helpful to select candidate genes potentially involved in different horticultural traits, such as flowering, floral architecture, scent production and emission, senescence and abscission. We used the microarray developed herein to identify genes whose expression is associated with some of these rose important traits, such as flower initiation, development and senescence. Rosa1_Affyarray harbors sequences from ESTs found in petals of different rose genotypes [5], [14] (http://urgi.versailles.inra.fr/GnpSeq) and thus may be helpful to identify genes associated with other rose traits such as scent biosynthesis and/or emission genes. The rose is among the species that exhibit the highest scent complexity [47]–[48] [12] and some scent biosynthesis pathways are unique to the rose or not yet identified in other model species including other members of the Rosaceae genus [11], [49]. QTLs have been identified to be associated to several important traits of the rose [50]. However, the heterozygous genome of the rose complicates the breeding programs to select for several traits simultaneously. The identification of genes whose expression correlates with important ornamental traits can facilitate and accelerate candidate gene identification for rose breeding by marker assisted selection or genomic selection. For example, this dataset can provide researchers with a useful resource on the expression of candidate genes within a given mapping interval. Furthermore, the rapidly progressing high throughput sequencing technologies should allow the generation of precise genetic maps for the rose that could be combined to refined transcriptomics approaches to identify the genes responsible for important horticultural traits in the rose, and allow subsequent marker-assisted selection.

## Materials and Methods

### Plant material

*R. wichurana* was obtained from ‘Jardin de Bagatelle’ (Paris, France) and *R. x hybrida* cv. Félicité et Perpétue from the Loubert Nursery (Rosier sur Loire, France). Plants were grown outdoors on their own roots as previously described [13]. In spring, at different time points (see results), terminal parts of the growing shoot were harvested and partly dissected (removal of young leaves). *R. chinensis* cv. Old Blush was propagated by cuttings from the Lyon Botanical Garden. Plants were grown in the greenhouse with 16 h/8 h day/night and 25°C/14°C day/night temperature. No specific permits were required for the described filed studies, no specific permissions were required for these locations, the location is not privately owned or protected, and the field studies did not involve endangered or protected species.

### Light microscopy and SEM imaging of meristems and early flower development

Samples were dissected under a binocular stereomicroscope and then fixed in 4% glutaraldehyde (v/v) in 0.1 M phosphate buffer (pH 7.2) for 2 h at 4°C under vacuum. Samples were dehydrated in a graded ethanol series and embedded in Technovit 7100 [51]. Sections of 1.5 to 2.0 µm (Leica RM 2165 microtome) were stained with toluidine blue and examined under an Olympus BH2-RFC microscope coupled to a 3CCD Sony camera.

For scanning electron microscopy, terminal part of the shoot was carefully dissected. After a fixation in 4% glutaraldehyde (v/v), followed by post-fixation with osmium tetroxide, the sample was dehydrated in a graded alcohol series and in acetone. Dehydration was completed by critical point drying. Sample were then coated with gold (MED 020 BALTEC) and observed with a JEOL JSM-63017 scanning electron microscope.

### RNA samples preparation

Two independent biological replicates were produced for each samples at different stages. For each biological repetition and each point, RNA samples were obtained by pooling vegetative or floral tissue from at least five different plants. For *R. chinensis* cv. Old Blush samples, meristems or flowers were dissected and collected individually on plants at developmental growth stages, cultivated in greenhouse conditions as previously described [55]. For *R. wichurana* and *R. x hybrida* cv. Félicité et Perpétue, RNA was extracted from non-dissected buds, including either the vegetative meristem and its surrounding leaves or the pre-floral/floral meristem and its surrounding leaves and bracts.Total RNA was extracted using RNeasy Plant Mini Kit (Qiagen) according to the supplier's instructions.

### AFFYMETRIX Array hybridization

RNA samples were checked for their integrity on The Agilent 2100 bioanalyzer according to the Agilent Technologies (Waldbroon, Germany).

Two µg of total RNA were used to synthesize biotin-labeled cRNAs with the One-cycle cDNA synthesis kit (Affymetrix, Santa Clara, CA). Superscript II reverse transcriptase and T7-oligo (dT) primers were used to synthesize the single strand of cDNA at 42°C during 1 hour followed by the synthesis of the double stranded cDNA by using DNA ligase, DNA polymerase I and RNaseH during 2 hours at 16°C. Clean up of the double-stranded cDNA was performed with Sample Cleanup Module (Affymetrix) followed by *in vitro* transcription (IVT) in presence of biotin-labeled UTP using GeneChip® IVT labelling Kit (Affymetrix). Quantity of the labelled-cRNA with RiboGreen® RNA Quantification Reagent (Turner Biosystems, Sunnyvale, CA) was determined after cleanup by the Sample Cleanup Module (Affymetrix). Fragmentation of 10 µg of labelled-cRNA was carried out for 35 minutes at 94°C, followed by hybridization during 16 hours at 45°C to Affymetrix GeneChip® Rosa1 Genome Array representing approximately 4869 genes. After hybridization, the arrays were washed with 2 different buffers (stringent: 6× SSPE, 0.01% Tween-20 and non-stringent: 100 mM MES, 0.1 M [Na+], 0.01% Tween-20) and stained with a complex solution including Streptavidin R-Phycoerythrin conjugate (Invitrogen/molecular probes, Carlsbad, CA) and anti Streptavidin biotinylated antibody (Vectors laboratories, Burlingame, CA). The washing and staining steps were performed in a GeneChip® Fluidics Station 450 (Affymetrix). The Affymetrix GeneChip® Rosa1 Genome Arrays were finally scanned with the GeneChip® Scanner 3000 7G piloted by the GeneChip® Operating Software (GCOS).

### Statistical Analysis of Microarray Data

The data were normalized with the gcrma algorithm [52], available in the Bioconductor package [53]. To determine differentially expressed genes, we performed a usual two group t-test that assumes equal variance between groups. The variance of the gene expression per group is a homoscedastic variance, where genes displaying extremes of variance (too small or too large) were excluded. The raw P values were adjusted by the Bonferroni method, which controls the Family Wise Error Rate (FWER) [54]. A gene is declared differentially expressed if the Bonferroni P-Value is less than 0.05.

### Data Deposition

All this steps were performed on Affymetrix plateform at INRA-URGV, Evry. The raw. CEL files were imported in R software for data analysis. All raw and normalized data are available through the CATdb database (AFFY_PetalDvt_Lyon_Rose, [55]) and from the Gene Expression Omnibus (GEO) repository at the National Center for Biotechnology Information (NCBI) [56], accession number GSE18357.

### Validation of genes expression using quantitative real-time PCR

Only genes that were involved in floral development were analyzed for microarray data validation. One microgram total RNA (treated with DNAse) was used in a reverse transcription assay with RevertAid M-MuLV Reverse Transcriptase (Fermentas, Burlington, Ontario). Target cDNAs were quantified by qPCR using FastStart universal SYBR green master (Roche, Basel, Switzerland) on a Step-OnePlus Real-Time PCR System (Applied Biosystems, Foster City, CA USA). Expression levels were normalized with *RhαTubuline*, *RhGAPDH* and *RhEF1α* reference genes. These genes were validated as reference genes using the GeNorm application [57]. Three independent biological replicates (pools of dissected flowers from at least 5 different plants) were used for each experiment and two qPCR technical replicates were performed for each biological replicate. Primer sequences are available in Table S9. The correlation between the microarray results, and those obtained by qPCR was assessed by calculating the Pearson's product moment correlation coefficient [58], [59].

## Supporting Information

Figure S1 **Real time quantitative RT-PCR (qPCR) analysis of 18 selected differentially expressed genes during rose floral organogenesis and senescence in** ***R. chinensis*** **cv Old Blush.**
(TIFF)

Table S1 **Full array sequences annotation and ontology.**
(XLSX)

Table S2 **Genes differentially expressed during floral initiation in** ***R. wichurana*** **.**
(XLSX)

Table S3 **Genes differentially expressed during floral initiation in** ***R. x hybrida*** **cv. Félicité et Perpétue.**
(XLSX)

Table S4 **List of genes repressed (A) or activated (B) during flower initiation.**
(XLSX)

Table S5 **Microarray and qRT-PCR results of 25 selected genes with their replicate-level Pearson correlation.**
(DOCX)

Table S6 **Genes differentially expressed during early floral organogenesis in** ***R. chinensis*** **cv. Old Blush.**
(XLSX)

Table S7 **Genes differentially expressed during floral organ elongation in** ***R. chinensis*** **cv. Old Blush.**
(XLSX)

Table S8 **Genes differentially expressed during flower opening and senescence in** ***R. chinensis*** **cv. Old Blush.**
(XLSX)

Table S9 **Primers used in this study.**
(DOC)
