# Supplementary figures and images for: Genomic Approach to Study Floral Development Genes in Rosa sp
Source: PLoS One. 2011 Dec 14;6(12):e28455. doi: 10.1371/journal.pone.0028455 (PMC3237435; doi:10.1371/journal.pone.0028455)

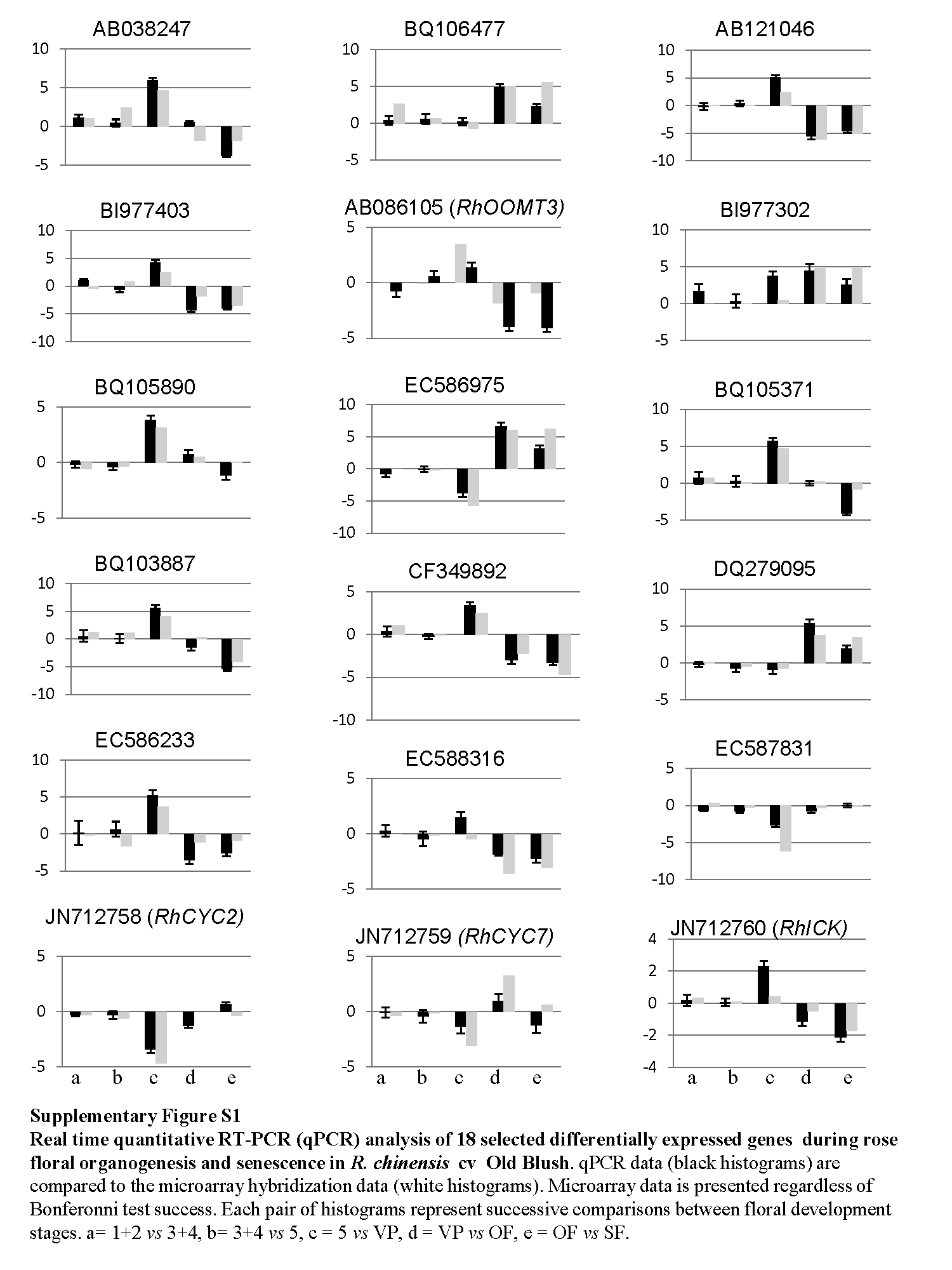

Supplement: Figure S1 — Real time quantitative RT-PCR (qPCR) analysis of 18 selected differentially expressed genes during rose floral organogenesis and senescence in R. chinensis cv Old Blush. (TIFF) [file pone.0028455.s001.tiff]
