# Supplementary material for: Genomic Approach to Study Floral Development Genes in Rosa sp
Source: PLoS One. 2011 Dec 14;6(12):e28455. doi: 10.1371/journal.pone.0028455 (PMC3237435; doi:10.1371/journal.pone.0028455)
Supplement: Table S5 — Microarray and qRT-PCR results of 25 selected genes with their replicate-level Pearson correlation. (DOCX) [file pone.0028455.s006.docx]

# Supplementary Table S5- Microarray and qRT-PCR results of 25 selected genes with their replicate-level Pearson correlation.

# EST Identifier Putative function Correlation p-value

AB025643 RhSHP 0.939 0.0181

AB025645 RhAG 0.810 0.0969

AB055966 *RhTM6/MASAKO B3*  0.265 0.6662

AB086105 RhOOMT3 0.816 0.0923

AB201048 Putative anthocyanidin 5,3-O-glucosyltransferase 0.961 0.0090

AF441282 Putative 1-aminocyclopropane-1-carboxylate oxidase 0.885 0.0462

BI977302 Putative bZip transcription factor 0.574 0.3116

BI977403 Senescence-associated protein SAG29 homologue 0.889 0.0437

BI978992 Putative NAM-like protein 0.960 0.0096

BQ103887 Ripening-related pectate lyase homologue 0.973 0.0052

BQ105371 Polygalacturonase inhibitor-like protein homologue 0.941 0.0170

BQ105890 Putative bHLH transcription factor 0.940 0.0174

CF349892 Endo-beta-1,4-glucanase precursor homologue 0.939 0.0178

DQ279095 xyloglucan endotransglucosylase hydrolase homologue 0.905 0.0344

EC586233 Putative Pollen coat protein-like 0.837 0.0773

EC586975 Putative Glycosyltransferase 0.938 0.0182

EC587831 Putative Chromomethylase 1 0.943 0.0161

BI978095 Putative MYB26 0.963 0.0084

AB038247 Putative flavonol synthase, 0.825 0.0856

BQ106477 RhWUSCHEL 0.765 0.1318

EC588316 Ripening regulated protein DDTFR18 homologue 0.829 0.0825

AB121046 Putative phloroglucinol O-methyltransferase 0.969 0.0064

JN712760 RhICK 0.848 0.0689

JN712758 RhCYC2 0.972 0.0056

JN712759 RhCYC7 0.789 0.1123
