# Supplementary material for: Genomic Approach to Study Floral Development Genes in Rosa sp
Source: PLoS One. 2011 Dec 14;6(12):e28455. doi: 10.1371/journal.pone.0028455 (PMC3237435; doi:10.1371/journal.pone.0028455)
Supplement: Table S9 — Primers used in this study. (DOC) [file pone.0028455.s010.doc]

Supplemental Table S9 : Primers used for the qPCR experiments

| Gene code | Primer name | Primer sequence |
| --- | --- | --- |
| *AB025643* | AB025643_Q3 | TTTTCGATCCTCTTGATCTCG |
|  | AB025643_Q5 | CCTGAGAGCTCTTCCCAAAA |
| *AB025645* | AB025645_Q3 | ACCCCTGTTACTGTTCTGCAA |
|  | AB025645_Q5 | CAGCAAGAAGCTGCCAAAC |
| *AB038247* | AB038247_Q3b | GGATTCTTAGGCCAAAAGCAG |
|  | AB038247_Q5b | GAAAGGGTGGGTGGATCAT |
| *AB086105* | AB086105_Q3 | GGGTAGGCCTCAATAATGGAC |
|  | AB086105_Q5 | CAGGCTTCCCTCGTTACAAA |
| *AB121046* | AB121046_Q3 | GCCCTTAATGTGCGGGTA |
|  | AB121046_Q5 | GGGAGGTGGAATTGGTGTTA |
| *AB201048* | AB201048_Q3 | AACCCGGTTTCGGACTCTT |
|  | AB201048_Q5 | GGTGTTTCTGGTGGAGGAGAT |
| *BI977302* | BI977302_Q3 | CGAGCCGATTCTCTGTTTG |
|  | BI977302_Q5 | GCAGCTGGGCAACATCAT |
| *BI977403* | BI977403_Q3 | TGCTTGAAACCCTTCAGTTGT |
|  | BI977403_Q5 | GGCAACATTATCTCCTTTATGGTT |
| *BI978095* | BI978095_Q3 | GGGCATAAAGGGTAGGTGGT |
|  | BI978095_Q5 | AATGGGATCGAGCAGCAC |
| *BI978992* | BI978992_Q3 | CTAGCTCCGTTCGGGTACTTC |
|  | BI978992_Q5 | GGGGAGCAGGAGTGGTATTT |
| *BQ103887* | BQ103887_Q3 | ATTCGAGAGCGAGTTGTGGT |
|  | BQ103887_Q5 | GATGGGGATGGTATATCCATTTT |
| *BQ105371* | BQ105371_Q3 | CTCCTGGTCGCAGCTGAT |
|  | BQ105371_Q5 | GGGAATTTTCAAATCATGGACA |
| *BQ105890* | BQ105890_Q3 | TGGCTGGCTCAGAAGGTTTA |
|  | BQ105890_Q5 | CAAGTTCAGGGGCTGATGAC |
| *BQ106477* | BQ106477_Q3b | AACCCAGCAACAAATGGAAT |
|  | BQ106477_Q5b | TACTACTCCCGGCTGATTGG |
| *CF349892* | CF349892_Q3 | AAGCTGGTTATAGCCGCACT |
|  | CF349892_Q5 | GCTCCTCTTATCGGTTTATTGG |
| *DQ279095* | DQ279095_Q3 | ATTGGAAACCCGTACCTGTG |
|  | DQ279095_Q5 | ACAATGGTGGCAATGAGATTC |
| *EC586233* | EC586233_Q3 | TGCCAGTAGCATTCTTGACG |
|  | EC586233_Q5 | GCCAAGCTATTCAGCAAACG |
| *EC586975* | EC586975_Q3 | GTGGCATCTTCATCCGATTC |
|  | EC586975_Q5 | GCTTCCCTGATTTTCGGTTC |
| *EC587831* | EC587831_Q3 | TCAAAACATCCGTTAGGCAAA |
|  | EC587831_Q5 | GGTTCTGCTGGTGAGGATTC |
| *EC588316* | EC588316_Q3b | GCAATGGGAACATAATTGCAT |
|  | EC588316_Q5b | AATCGGGCTGACAGATGAAG |
| *JN712760* | ICKqF3 | aatcctcactgcttcttcgcc |
|  | ICKqR1 | tgtagacggtttcacaactc |
| *JN712758* | Y2qF52 | acacccaagcagttgttcctc |
|  | Y2qR5 | gttcccattgttcttctgtgca |
| *JN712759* | Y7qF5 | aatgcccaaaggaatctgag |
|  | Y7qR5 | attcttcatcctcctccatcc |
| *EC589884* | RhGAPDH_QS1 | gctggcaggtatcctttctg |
|  | RhGAPDH_QAS1 | ggcgacaatatcagccaagt |
| *BI978089* | RhEF1-QS1 | GGGTAAGGAGAAGGTTCACATC |
|  | RhEF1-QAS1 | CAGCCTCCTTCTCAAACCTCT |
| *BI978618* | RhTCTP-R2 | CTTGGTTGCTCCCTCAATGT |
|  | RhTCTP-F2 | GATGCTGATGAGGGTGTTGA |
| *AF394915* | *RhαTub_QS1* | ATTGAGCGTCCCACCTACAC |
|  | *RhαTub_QAS1* | AGCATGAAATGGATCCTTG |
